# Supplementary material for: Comparing Inflammatory Biomarkers in Cardiovascular Disease: Insights from the LURIC Study
Source: Int J Mol Sci. 2025 Jul 29;26(15):7335. doi: 10.3390/ijms26157335 (PMC12347170; doi:10.3390/ijms26157335)
Supplement: Supplementary file 1 [file ijms-26-07335-s001.zip › ijms-3706325-supplementary.pdf]

Supplementary material

Supplementary Table S1: Association of **hsCRP (>3 mg/L) and SAA (>6.4 mg/L)** concentrations with all-cause and cardiovascular mortality based on established clinical cut-offs.[1]

	**All-cause mortality**		**Cardiovascular mortality**	
	**HR (95%CI)**	**P-Value**	**HR (95%CI)**	**P-Value**
Crude				
both low	**1**	**-**	**1**	**-**
hsCRP high	1.50(1.26-1.80)	<0.001	1.34(1.06-1.70)	0.015
SAA high	0.98(0.72-1.34)	0.917	0.89(0.59-1.35)	0.585
both high	1.87(1.62-2.17)	<0.001	2.05(1.71-2.46)	<0.001
Model 1				
both low	1	-	1	-
hsCRP high	1.41(1.18-1.68)	<0.001	1.26(0.99-1.60)	0.057
SAA high	0.98(0.71-1.34)	0.893	0.88(0.58-1.35)	0.566
both high	1.72(1.49-1.99)	<0.001	1.89(1.57-2.27)	<0.001
Model 2				
both low	1	-	1	-
hsCRP high	1.12(0.92-1.37)	0.255	0.98(0.75-1.27)	0.865
SAA high	0.78(0.55-1.10)	0.150	0.73(0.47-1.14)	0.168
both high	1.20(1.02-1.42)	0.031	1.30(1.06-1.61)	0.014
Model 3				
both low	1	-	1	-
hsCRP high	0.629	1.05(0.86-1.28)	0.92(0.71-1.20)	0.555
SAA high	0.078	0.73(0.52-1.04)	0.69(0.44-1.09)	0.110
both high	0.773	1.03(0.86-1.23)	1.13(0.90-1.43)	0.284

[1] Model 1 was adjusted for age and sex. Model 2 extended this by including body mass index (BMI), low-density lipoprotein cholesterol (LDL-C), high-density lipoprotein cholesterol (HDL-C), albumin, medication (lipid lowering therapy, diuretics, antiplatelet drugs, coumarins, calcium antagonists, AT2 receptor antagonists, cortisol, betablocker) and stratification for the presence of diabetes mellitus, hypertension, smoking status and estimated glomerular filtration rate (eGFR). Model 3 was Model 2 + (log) IL-6.

Supplementary Table S2: Association **of IL-6 (7pg/ml) and SAA (>6.4 mg/L)** concentrations with all-cause and cardiovascular mortality based on established clinical cut-offs.[1]

	**All-cause mortality**		**Cardiovascular mortality**	
	**HR (95%CI)**	**P-Value**	**HR (95%CI)**	**P-Value**
Crude				
both low	**1**	-	**1**	-
Il-6 high	1.61(1.24-2.09)	<0.001	1.57(1.11-2.22)	0.010
SAA high	1.25(1.07-1.46)	0.004	1.41(1.16-1.71)	0.001
both high	2.22(1.89-2.60)	0.000	2.47(2.03-3.02)	<0.001
Model 1				
both low	**1**	-	**1**	-
IL-6 high	1.41(1.09-1.84)	0.010	1.38(0.98-1.95)	0.066
SAA high	1.28(1.10-1.50)	0.002	1.44(1.19-1.75)	<0.001
both high	1.80(1.53-2.12)	0.000	2.01(1.65-2.46)	<0.001
Model 2				

both low	**1**	-	**1**	-
IL-6 high	1.22(0.93-1.61)	0.158	1.17(0.81-1.70)	0.398
SAA high	1.02(0.86-1.21)	0.780	1.17(0.94-1.45)	0.151
both high	1.27(1.06-1.53)	0.011	1.41(1.12-1.78)	0.004
Model 3				
both low	**1**	-	**1**	-
IL-6 high	1.18(0.89-1.56)	0.250	1.13(0.78-1.64)	0.518
SAA high	0.90(0.74-1.09)	0.268	1.03(0.80-1.31)	0.841
both high	1.00(0.78-1.28)	0.988	1.12(0.82-1.53)	0.475

[1] Model 1 was adjusted for age and sex. Model 2 extended this by including body mass index (BMI), low-density lipoprotein cholesterol (LDL-C), high-density lipoprotein cholesterol (HDL-C), albumin, medication (lipid lowering therapy, diuretics, antiplatelet drugs, coumarins, calcium antagonists, AT2 receptor antagonists, cortisol, betablocker) and stratification for the presence of diabetes mellitus, hypertension, smoking status and estimated glomerular filtration rate (eGFR). Model 3 was Model 2 + (log) hsCRP.

Supplementary Table S3: Association of **hsCRP (>3 mg/L) and IL-6 (7 pg/ml)** concentrations with all-cause and cardiovascular mortality based on established clinical cut-offs..[1]

	**All-cause mortality**		**Cardiovascular mortality**	
	**HR(95%CI)**	**P-Value**	**HR(95%CI)**	**P-Value**
Crude				
both low	**1**	**-**	**1**	**-**
IL6 high	0.96(0.63-1.47)	0.856	0.97(0.56-1.66)	0.900
hsCRP high	1.41(1.22-1.64)	<0.001	1.46(1.21-1.77)	<0.001
both high	2.53(2.15-2.97)	<0.001	2.68(2.19-3.28)	<0.001
Model 1				
both low	**1**	-	**1**	-
IL6 high	0.90(0.59-1.37)	0.616	0.90(0.53-1.55)	0.716
hsCRP high	1.39(1.20-1.62)	<0.001	1.44(1.19-1.75)	<0.001
both high	2.02(1.72-2.37)	<0.001	2.15(1.75-2.63)	<0.001
Model 2				
both low	**1**	-	**1**	-
IL6 high	0.79(0.51-1.23)	0.293	0.76(0.43-1.35)	0.349
hsCRP high	1.09(0.92-1.29)	0.300	1.11(0.90-1.37)	0.332
both high	1.41(1.17-1.70)	<0.001	1.45(1.15-1.84)	0.002
Model 3				
both low	**1**	-	**1**	-
IL6 high	0.79(0.51-1.23)	0.295	0.76(0.43-1.34)	0.341
hsCRP high	1.11(0.92-1.32)	0.272	1.04(0.83-1.31)	0.725
both high	1.44(1.14-1.82)	0.002	1.27(0.95-1.71)	0.109

[1] Model 1 was adjusted for age and sex. Model 2 extended this by including body mass index (BMI), low-density lipoprotein cholesterol (LDL-C), high-density lipoprotein cholesterol (HDL-C), albumin, medication (lipid lowering therapy, diuretics, antiplatelet drugs, coumarins, calcium antagonists, AT2 receptor antagonists, cortisol, betablocker) and stratification for the presence of diabetes mellitus, hypertension, smoking status and estimated glomerular filtration rate (eGFR). Model 3 was Model 2 + log(SAA).

Supplementary Table S4: Association of hsCRP (>3mg/L), SAA (>6.4mg/L) and IL-6 (7pg/ml) concentrations with all-cause and cardiovascular mortality

	All-cause mortality		Cardiovascular mortality	
	**HR (95%CI)**	**P-Value**	**HR (95%CI)**	**P-Value**
Crude				
all low	**1**	-	**1**	-
1-2 high	1.36(1.15-1.61)	<0.001	1.24(1.00-1.54)	0.054
all high	1.89(1.63-2.20)	<0.001	2.08(1.73-2.51)	<0.001
Model 1				
all low	**1**	-	**1**	-
1-2 high	1.29(1.09-1.53)	0.003	1.17(0.94-1.46)	0.152
all high	1.74(1.49-2.02)	<0.001	1.91(1.58-2.30)	<0.001
Model 2				
all low	**1**	-	**1**	-
1-2 high	1.03(0.86-1.24)	0.722	0.93(0.73-1.18)	0.552
all high	1.20(1.01-1.43)	0.037	1.31(1.05-1.62)	0.015

[1] Model 1 was adjusted for age and sex. Model 2 extended this by including body mass index (BMI), low-density lipoprotein cholesterol (LDL-C), high-density lipoprotein cholesterol (HDL-C), albumin, medication (lipid lowering therapy, diuretics, antiplatelet drugs, coumarins, calcium antagonists, AT2 receptor antagonists, cortisol, betablocker) and stratification for the presence of diabetes mellitus, hypertension, smoking status and estimated glomerular filtration rate (eGFR).

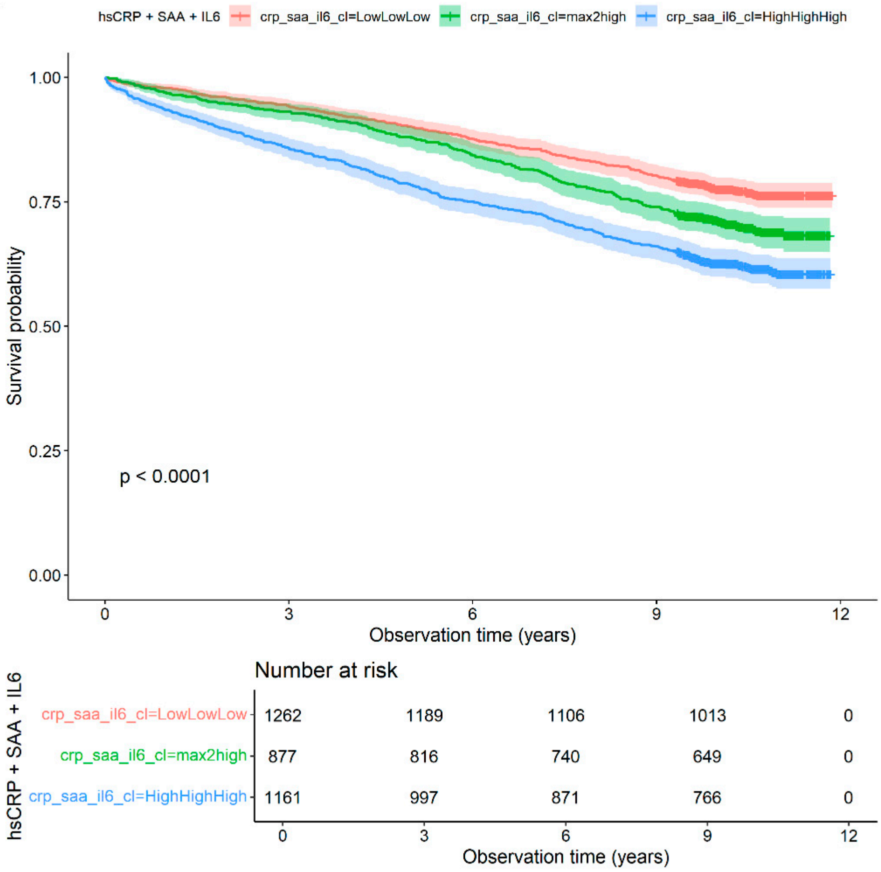

Supplementary Figure SF1: Kaplan–Meier survival analysis comparing participants with all three inflammatory markers above the clinical threshold (N = 1161), those with all markers below the threshold (N = 1262), and those with only one or two elevated markers (N = 877).
